# Baclofen but Not Diazepam Alleviates Alcohol-Seeking Behavior and Hypothalamic–Pituitary–Adrenal Axis Dysfunction in Stressed Withdrawn Mice

**DOI:** 10.3389/fpsyt.2019.00238

**Published:** 2019-04-16

**Authors:** Yolaine Rabat, Nadia Henkous, Marc Corio, Xavier Nogues, Daniel Beracochea

**Affiliations:** ^1^Université de Bordeaux, Institut de Neurosciences Cognitives et Intégratives d’Aquitaine (INCIA), CNRS UMR 5287, Pessac, France; ^2^Université de Bordeaux, Bordeaux, France

**Keywords:** gamma-aminobutyrique acid (GABA), ethanol, anxiety, glucocorticoids, corticosterone, benzodiazepines, dependence

## Abstract

This study compares the impact of repeated injections of baclofen (an agonist of GABA_B_ receptors) or diazepam (a benzodiazepine having an agonist action on GABA_A_ receptors) given during the alcohol-withdrawal period on the stress-induced restoration of alcohol-seeking behavior and hypothalamic–pituitary–adrenal (HPA) axis dysfunction after a long (4 weeks) abstinence. Thus, C57BL/6 mice were submitted to a 6-month alcohol consumption [12% volume/volume (v/v)] and were progressively withdrawn to water before testing. Diazepam (Valium^®^, Roche) and baclofen (Baclofen^®^, Mylan) were administered intraperitoneally for 15 consecutive days (1 injection/day) during the withdrawal period at decreasing doses ranging from 1.0 mg/kg (Day 15) to 0.25 mg/kg (Day 1) for diazepam and from 1.5 mg/kg (Day 15) to 0.37 mg/kg (Day 1) for baclofen. Alcohol-seeking behavior was evaluated by alcohol-place preference in an odor recognition task. In the stress condition, mice received three electric footshocks 45 min before behavioral testing. Blood was sampled immediately after behavioral testing, and plasma corticosterone concentrations were measured by commercial enzyme immunoassay kits. Results showed that non-stressed withdrawn mice did not exhibit alcohol-place preference or alteration of plasma corticosterone concentrations relative to water controls. After stress, however, withdrawn mice exhibited a significant alcohol-place preference and higher circulating corticosterone concentrations as compared to stressed water controls. Interestingly, repeated administration during the withdrawal phase of baclofen but not diazepam suppressed both the alcohol-place preference and normalized corticosterone levels in stressed withdrawn animals. In conclusion, this study evidences that a pre-treatment with baclofen but not with diazepam during the withdrawal phase normalized, even after a long period of abstinence, the HPA axis response to stress, which contributes to the long-term preventing effects of this compound on alcohol-seeking behavior.

## Introduction

There is substantial evidence that cognitive and neurobiological alterations are either dramatically enhanced or gradually developed after alcohol withdrawal (1–4). One of the main disturbances associated with alcohol withdrawal involves a dysregulation of the hypothalamic–pituitary–adrenal (HPA) axis, which accounts for excessive glucocorticoid (GC) release (5). Thus, evidence in humans (6–8) and rodents (9–11) has shown that alcohol withdrawal markedly affects plasma GC levels. Moreover, studies in rodents have evidenced brain regional GC disturbances after long alcohol withdrawal periods (12), which account for protracted cognitive dysfunction (13, 14).

The relationships between the HPA axis activity, craving, and alcohol intake during early abstinence have been well documented (15, 16). Clinical and experimental studies pointed out how corticosterone and stress interact with the brain reward system and contribute to alcohol reinforcing effects (17, 18) and relapse to alcohol-seeking behavior (15). Therefore, reducing or suppressing GC dysregulations in alcohol-withdrawn mice can prevent relapse to alcohol-seeking behavior.

A way to alleviate the HPA axis dysregulation is to act on the GABAergic (gamma-aminobutyrique acid) neurotransmission (19, 20). Indeed, baclofen (an agonist of GABA_B_ receptors) and diazepam (a benzodiazepine having an agonist action on GABA_A_ receptors) have been found to reduce the HPA axis activity in withdrawn alcoholics (7, 21–24) and addiction to alcohol in both humans and animals (25–30). Main issues remain, however, i) to determine the beneficial effects of these compounds after a long abstinence, since most of the existing studies have been mainly carried out after short withdrawal periods (31, 32), and ii) since stress is a main factor of alcohol-seeking behavior and relapse (33, 34), if the long-term beneficial effects of these compounds are still observed in the stress condition.

We previously developed a mouse model of alcohol withdrawal inducing protracted cognitive deficits and persistent brain regional GC alterations up to 6 weeks after the cessation of alcohol intake (13, 14, 35). As yet, however, we did not investigate if withdrawn mice exhibit persistent altered motivation for alcohol. Indeed, even though placed under a forced choice exposure, the C57BL/6 mice used in our previous studies are an alcohol-preferring strain, which have been found to consume a high daily amount of alcohol (36). Thus, we investigated in the present study alcohol-seeking behavior after a 1 week or 4 weeks’ cessation of alcohol intake in reference (non-stress) condition, or after the onset of a stressor, and the relative efficacy of diazepam and baclofen to counteract the long-term withdrawal-induced motivational deficits.

To that aim, we first determined the emotional profile (anxiety and depression-like behaviors) of withdrawn animals after short (1 week) or long (4 weeks) withdrawal periods. Then, in Experiment 2, we studied alcohol-seeking behavior in an odor recognition task and measured the circulating corticosterone concentrations in non-stressed or stressed mice after the 1-week or 4-week alcohol-withdrawal periods. Finally, in Experiment 3, we compared the effects of repeated injections of diazepam or baclofen delivered during the withdrawal phase on the stress-induced alcohol-seeking behavior and HPA axis dysfunction found in Experiment 2, more particularly in 4-week withdrawn animals.

## Materials and Methods

### Animals

Animals were male mice of the C57BL/6 strain (Charles River, L’Arbresle, France). They were housed by groups of 10 in collective cages (425 mm × 276 mm × 153 mm; 820 cm^2^) until they were 10 months old, in a temperature-controlled colony room (22 ± 1 C), under a 12:12 light–dark cycle (lights on at 7:00 a.m). They were provided with food and water or alcohol *ad libitum*. All procedures were carried out during the light phase of the cycle. Two weeks before the experiments, they were housed individually (331 mm × 159 × mm 132 mm; 335 cm^2^).

All experimental procedures were performed between 8:00 and 12:00 a.m. to prevent any circadian rhythm side effects on GC levels and were conducted in accordance with the European Union Directive 2010/63/European Union for animal experiments and local ethical committee (#5012089).

### Alcohol Administration and Withdrawal Procedures

The procedure has been described in full previously (13). Four-month-old mice were given, as their sole liquid source, water containing increasing concentrations of ethanol (from ethanol 95%; Prochilab, France) as follows: 4% (v/v) the first week, 8% (v/v) the second week, and 12% (v/v) for six consecutive months. The alcohol-preferring C57BL/6 mice have been chosen to reduce eventual stress-related exposure of alcohol, which has been found to be aversive in other strains of mice (36). Alcohol consumption was measured during the 6-month alcohol consumption period, by scoring each week the decrease of liquid consumption on graduated bottles. The mean daily alcohol intake per mouse was then calculated for each group of alcohol-withdrawn mice used in the study. At the end of that period, all mice were housed in individual cages and alcohol-treated mice were progressively withdrawn from alcohol. To that aim, alcohol was progressively replaced by water to avoid abrupt withdrawal symptoms as follows: 8% (v/v) for 3 days, 4% (v/v) for the next 3 days, and then water until the end of the experiments. Behavioral testing began either after 1 week (withdrawn 1W) or 4 weeks (withdrawn 4W) of water supply. To avoid potential negative effects of isolation in individual cages, wooden marbles were added in the cages that were constructed of transparent Plexiglas, allowing us to visualize congeners.

In the present study, 138 alcohol-withdrawn mice were attributed to 13 different independent groups over the three experiments of the study. Each task and experiment was performed with independent cohorts of mice. Thus, withdrawn 1W and withdrawn 4W mice belong to independent groups. A total of 154 mice were used as control mice, also attributed to independent groups. They were housed similarly as the alcohol-withdrawn groups, but permanently received water (control 1W and control 4W).

### Behavioral Tasks

**Figure 1:**
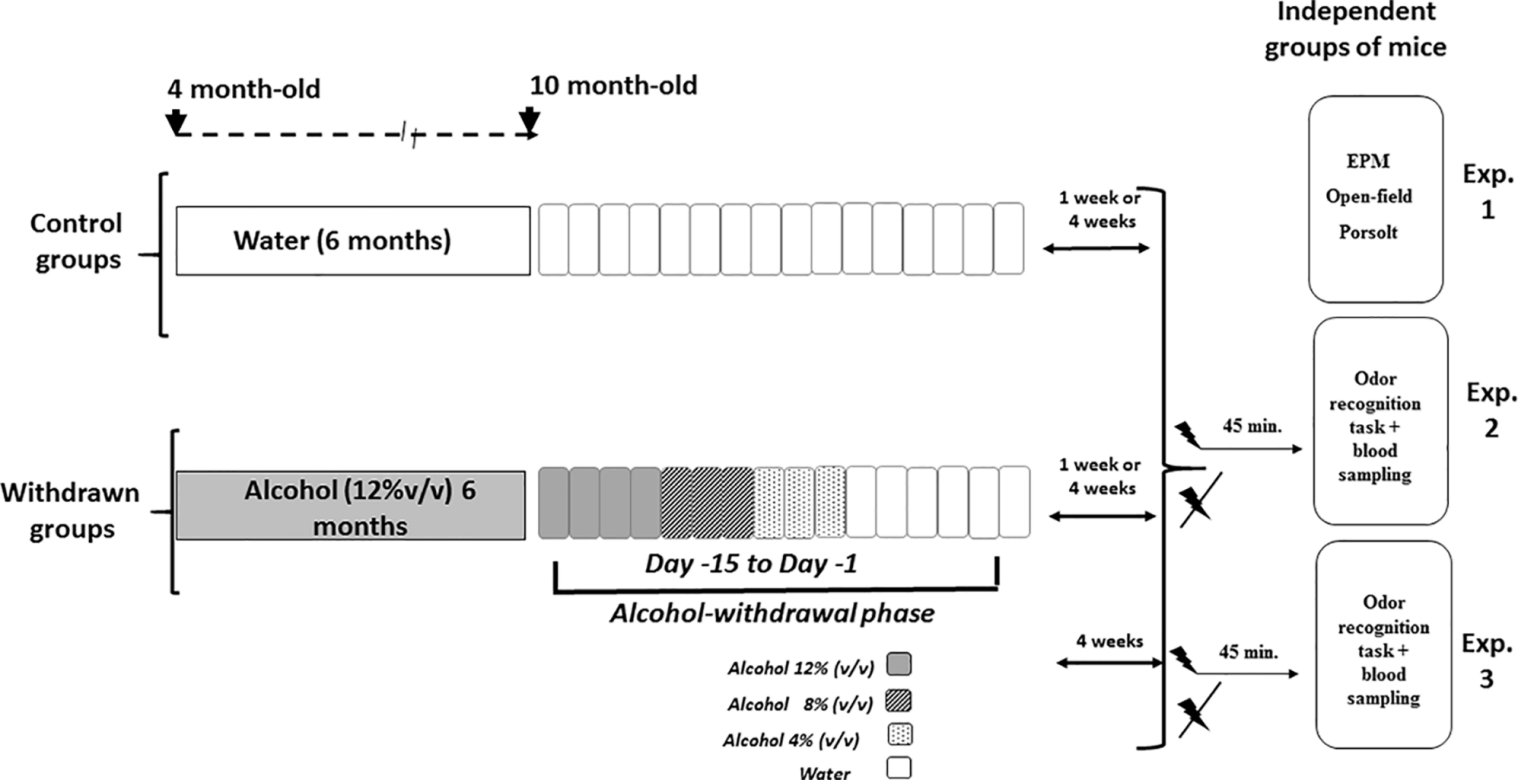
Schematic overview of the alcohol exposure, withdrawal, and behavioral experiments. The withdrawn groups were submitted to a 6-month exposure to alcohol (12% v/v) followed by a withdrawal phase that lasted 15 days (from Day 15 to Day 1), during which alcohol was progressively withdrawn from the solution by steps of 4% (Day 15 to Day 12: alcohol 12%; Day 11 to Day 9: alcohol 8% v/v; Day 8 to Day 6: alcohol 4%; then water for the remaining days). Control groups were exposed to the same general schedule except that they received permanently water as the sole source of fluid. Either 1 week or 4 weeks after the end of the withdrawal phase, independent groups of mice were used to test alcohol withdrawal anxiety-like reactivity (Experiment 1) in elevated plus maze (EPM), open-field, and Porsolt tasks; similarly, independent groups of 1-week or 4-week withdrawn mice were submitted to the odor recognition task and blood sampling in stress or non-stress conditions (Experiment 2 and Experiment 3: withdrawn 4-week mice only). The acute stress was constituted by electric footshocks delivered 45 min before behavioral testing.

Animals were submitted to behavioral testing either 1 week or 4 weeks after the end of the withdrawal periods and were therefore 9 to 10 months old at the time of behavioral testing. All behavioral tasks were videotaped (ViewPoint^®^, France) and analyzed blind to avoid any eventual biases. Experiments 1, 2, and 3 were run using different cohorts of mice. **Figure 1** depicts the general procedure used for alcohol administration and withdrawal, followed by behavioral testing of Experiments 1, 2, and 3.

#### Experiment 1: Evaluation of Emotional Reactivity

Each task was performed with different cohorts of mice.

##### The Elevated Plus Maze

This task is classically used to evaluate anxiety-like reactivity in rodents (37). This experiment was carried out on four independent groups of mice: control 1W (*N* = 12), withdrawn 1W (*N* = 11), control 4W (*N* = 12), and withdrawn 4W (*N* = 12).

The elevated plus maze was made of gray Plexiglas with four arms arranged in the shape of a plus sign. Each arm was 30 cm long, 7 cm wide, and elevated 40 cm above the ground. The four arms were joined at the center by a 7-cm square platform. Two opposite arms were “closed” by 17-cm-high side walls, while the other arms did not have side walls. Mice were allowed to freely explore all arms for 5 min. The “time” and “entry” ratios in the open arms (expressed in percentage) were used to measure anxiety-like behavior. Thus, the smaller these ratios are, the more “anxious-like” is the mouse.

##### The Open-Field Task

The open-field task allows the evaluation of anxiety-like locomotor reactivity in an open space, known to be an anxious situation in rodents (38). This experiment was carried out on four independent groups of mice: control 1W (*N* = 10), withdrawn 1W (*N* = 9), control 4W (*N* = 11), and withdrawn 4W (*N* = 12).

The open-field chamber was constructed of white Plexiglas in the shape of a circle measuring 100 cm in diameter and surrounded by a wall that is 15 cm high. The floor was made in white Plexiglas. A bright illumination was provided by two lamps positioned 2 m above the apparatus and providing a 600-lux illumination equally distributed over the whole surface of the apparatus. At the start of each trial, animals were placed in the periphery of the apparatus and the subjects were allowed to freely explore for 10 min. Two parameters were recorded: first, the latency to move from the periphery to the center; secondly, the total number of virtual crossed zones.

##### The Forced-Swim Test

The forced-swim test is commonly used to evaluate depressive-like behaviors in mice (39). This experiment was carried out on four independent groups: control 1W (*N* = 10), withdrawn 1W (*N* = 10), control 4W (*N* = 12), and withdrawn 4W (*N* = 12). This test consisted in laying down the animal in a cylindrical glass tank (Ø 15 cm) filled with water (30 cm high, 25 ± 1 C) for 6 min, during which the animal can swim, climb, or stay immobile. An animal presenting a depressive-like behavior will spend more immobility time than a non-depressive control mouse.

### Experiment 2: Alcohol-Place Preference and Hypothalamic–Pituitary–Adrenal Axis Activity After Short (1 Week) or Long (4 Weeks) Withdrawal Periods in Non-Stressed or Stressed Mice

The experiment was carried out on eight independent cohorts of mice, not used in Experiment 1: stressed control 1W (*N* = 9), stressed withdrawn 1W (*N* = 8), stressed control 4W (*N* = 8), stressed withdrawn 4W (*N* = 10), non-stressed control 1W (*N* = 9), non-stressed withdrawn 1W (*N* = 9), non-stressed control 4W (*N* = 9), and non-stressed withdrawn 4W (*N* = 8).

#### Odor Recognition Task

The odor of alcohol is used as a cue for inducing a place preference. In this task, mice were allowed to discriminate between an area humidified with water (neutral odor) and another one with the alcohol solution previously drunk during the 6-month alcohol exposure. The rationale is that the time spent in the alcohol area would be an index of a place preference and therefore of alcohol-seeking behavior.

##### Apparatus

**Figure 2:**
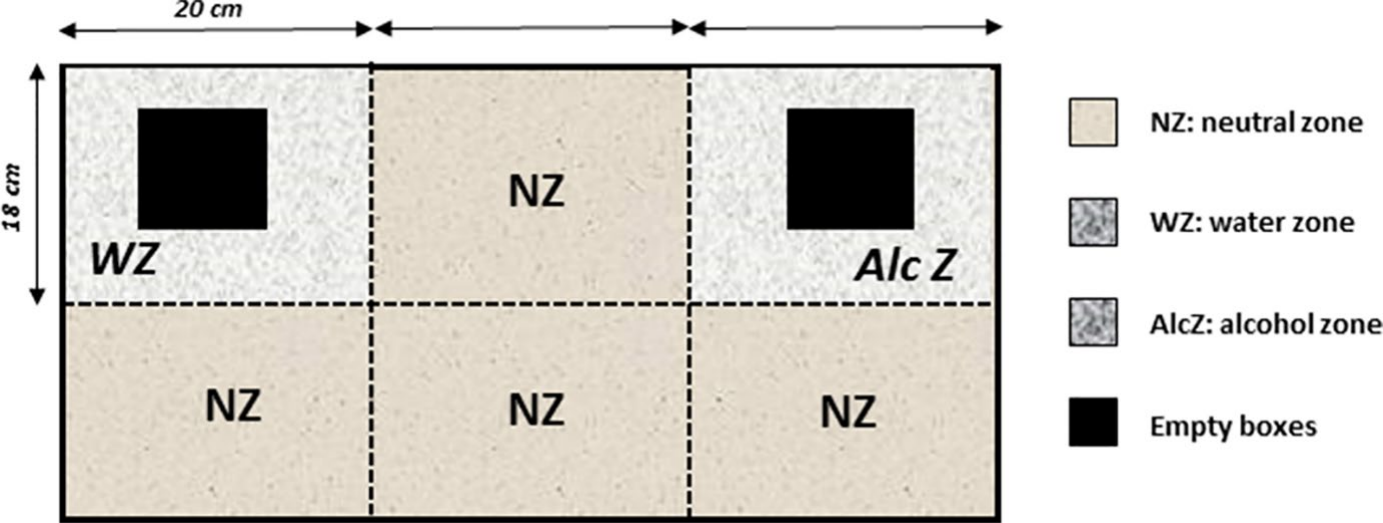
The odor recognition task. The apparatus is a rectangular chamber made of transparent Plexiglas. The floor was covered with sawdust and divided into separates zones that are wet either with water (water zone, WZ, neutral odor) or with a 12% alcohol solution (alcohol zone, AlcZ), with the remaining space being a neutral zone (NZ) covered only with dry sawdust. To enhance exploration, two identical familiar empty gray Plexiglas boxes were placed in the water and alcohol zones (empty boxes, black squares). The times spent exploring the water and alcohol zones were recorded, and a discrimination ratio was calculated (Time in AlcZ/Time in WZ).

It consisted of a rectangular chamber (60 cm long × 36 cm width × 20 cm high) made of transparent Plexiglas. The floor was covered with sawdust. Basically, mice were allowed to freely explore the apparatus containing two zones that are wet either with water (water zone: 20 cm × 18 cm) or with 12% alcohol (alcohol zone: 20 cm × 18 cm), with the remaining space being covered only with dry sawdust (neutral zone, NZ). To enhance exploration, two identical empty gray Plexiglas boxes (8 cm × 8 cm × 8 cm) were placed in the center of the water and alcohol zones. These boxes were previously placed in the mice’s home cage for 5 min during the 3 days preceding the odor recognition test, so that the object’s novelty cannot interfere with the preference for either zone (**Figure 2**).

##### Behavioral Protocol

During the recognition session, animals were placed in the central neutral zone and allowed to explore the apparatus for 6 min. The times spent exploring the alcohol and water zones were scored. A recognition index was calculated by the ratio: “Time in the Alcohol zone/Time in the Water zone.” A ratio equal to “1” means that both zones are equally explored, whereas a ratio above 1 indicates a preference for the alcohol zone.

##### Stress Delivery

Mice received electric footshocks 45 min before the recognition test, according to previous studies that have shown high plasma and brain corticosterone concentrations at this post-stress delay (40, 41). Stress was delivered in a chamber (20 cm × 15 cm × 15 cm). The floor consisted of 35 stainless steel rods (3 mm diameter), spaced 5 mm apart and wired to a shock generator for the delivery of three successive footshocks (0.9 mA; 1 s each) after 10, 30, and 50 s. Non-stressed mice were also placed in the chamber except that they did not receive footshocks.

#### Plasma Samples

Blood was collected in tubes containing 10% ethylenediaminetetraacetic acid (EDTA) by sub-mandibular sampling with 25-gauge needles after a very brief anesthesia (Isoflurane^®^; 30-s exposure before blood sampling). Sampling occurred between 08:00 a.m. and 12:00 p.m. on subgroups of mice chosen at random (*N* = 5) after behavioral testing. After 10 min of centrifugation at 3,000 rpm, plasma samples were stored at −80 C before analyses by a commercial enzyme immunoassay kit that allowed us to measure corticosterone concentrations (Correlate-EIATM, Assay Designs, Ann Arbor, MI).

### Experiment 3: Effects of Repeated Administration of Diazepam and Baclofen During Withdrawal on Alcohol-Place Preference

**Figure 3:**
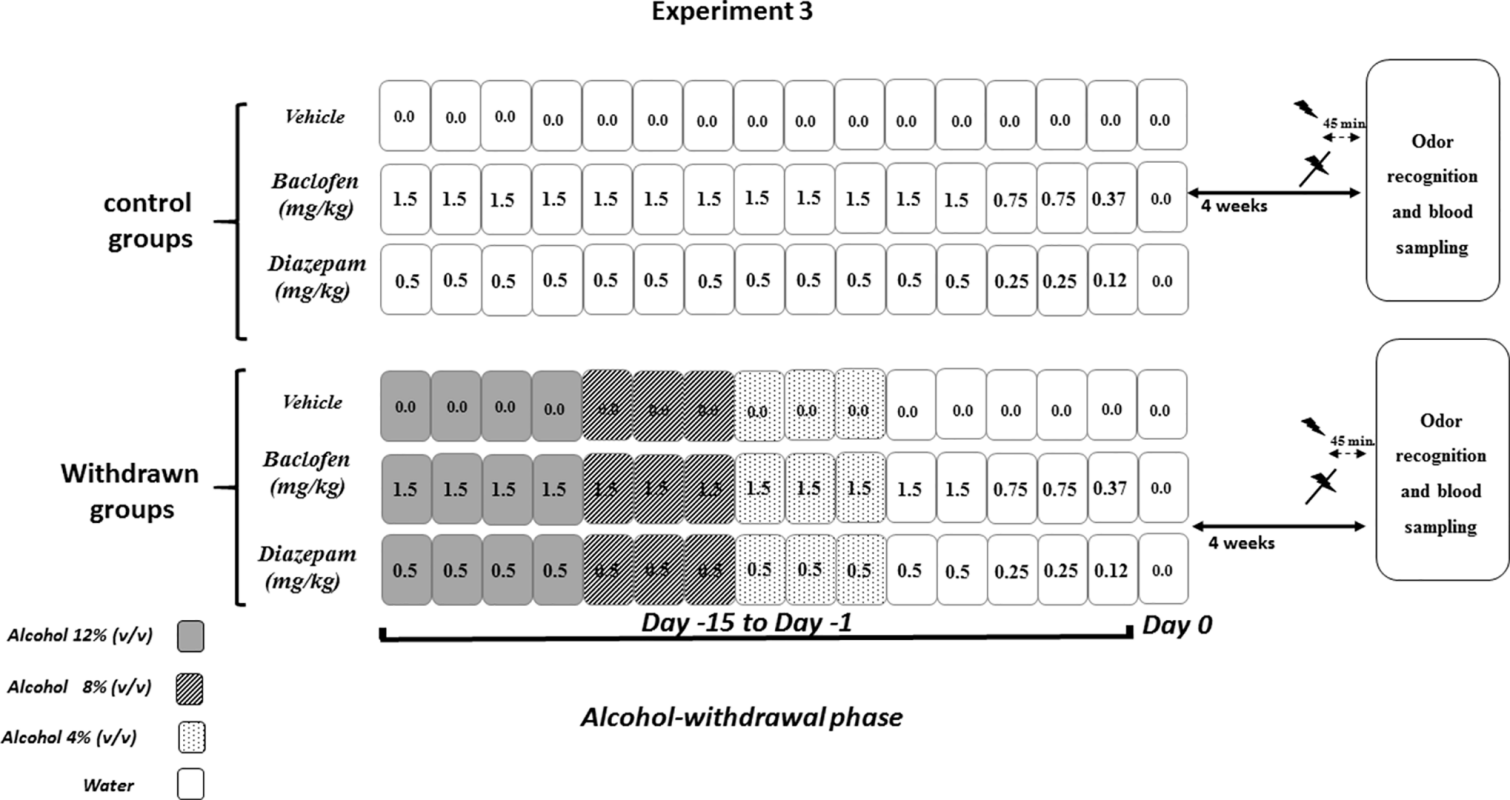
Pharmacological study (Experiment 3). Diazepam and baclofen were administered by Intraperitoneal injection (1/day) both in control (upper part) and in withdrawn mice (lower part, withdrawn groups) during 15 consecutive days (Day 15 to Day 1). The pharmacological treatments started when withdrawn mice were still under the alcohol regimen (12% v/v, dark rectangles), then 8% v/v (gray rectangles), and finally 4% v/v (light gray rectangles) followed by water (white rectangles). All mice received on the first 12 days of treatment either vehicles or diazepam at 0.5 mg/kg or baclofen at 1.5 mg/kg, followed at Days 3 and 2 by doses being half of the starting dose (0.25 mg/kg and 0.75 mg/kg, respectively, for diazepam and baclofen) and finally (Day 1) by 0.12 mg/kg or 0.37 mg/kg diazepam and baclofen doses, respectively. Water groups were submitted to the same pharmacological procedures as those used in withdrawn groups. Behavioral testing occurred 4 weeks after the last injection. The acute stress was constituted by electric footshocks delivered 45 min before behavioral testing.

All procedures were similar to those described in Experiment 2. This experiment was conducted in withdrawn 4W mice, which exhibited higher recognition scores in the odor recognition task. Experiment 3 was carried out on independent groups as follows: vehicle-withdrawn mice (*N* = 9 and *N* = 8 in stress and non-stress conditions, respectively), diazepam-treated withdrawn mice (*N* = 7 in both stress and non-stress conditions), and baclofen-treated withdrawn mice (*N* = 7 in both stress and non-stress conditions) were compared to control mice also submitted to repeated injections of either vehicle (*N* = 10 in both stress and non-stress conditions), diazepam (*N* = 8 in both stress and non-stress conditions), or baclofen (*N* = 9 and *N* = 8 in stress and non-stress conditions, respectively). The pharmacological administration procedure is depicted in **Figure 3**.

#### Repeated Diazepam and Baclofen Administration Procedures

Diazepam (Valium^®^, Roche) and baclofen (Baclofen^®^, Mylan) were diluted in saline (0.9% NaCl) and injected intraperitoneally (10 mL/kg, 1 injection/day). In all experiments, diazepam and baclofen administrations were performed on the 15 final days of the withdrawal phase when mice were still under a 12% ethanol (v/v) regimen.

Diazepam and baclofen were given during the 15 days preceding the end of the withdrawal phase (Day 0) (**Figure 3**). The doses were progressively decreased from Day 15 to Day 1, to avoid potential negative effects of an abrupt cessation of drug administrations. Diazepam was first delivered at a dose of 0.5 mg/kg for 12 consecutive days (Day 15 to Day 4), then at a dose of 0.25 mg/kg (Day 3 and Day 2), and finally at a dose of 0.12 mg/­kg (Day 1). Similarly, baclofen was first delivered at a dose of 1.5 mg/kg for 12 consecutive days (Day 15 to Day 4), then at a dose of 0.75 mg/kg (Day 3 and Day 2), and finally at a dose of 0.37 mg/(Day 1). Behavioral testing began 4 weeks after the last injection.

#### Measurement of Diazepam and Baclofen Concentrations in Blood

This study was designed to discard any acute effects of diazepam and baclofen on the alcohol-place preference scores. The concentrations in blood of baclofen and diazepam and of their active metabolites oxazepam and nordiazepam were determined by LC-MS/MS (liquid chromatography followed by double mass spectrometry) in independent groups of withdrawn mice at 3 delays (1 h, 24 h, and 48 h; *N* = 3 per time point in each group) after the last drug injections (Day 1 of the withdrawal phase). For that purpose, samples were sent to the Laboratory of Pharmacology and Toxicology (Bordeaux, France) for analyses.

### Statistical Analyses

Statistical analyses were performed using the Statview 5.0 software. Data were expressed as means ± SEM. Behavioral performance and corticosterone assays were analyzed using one- or two-way analyses of variance (ANOVAs). *Post hoc* Fisher Protected Least Significant Difference (PLSD) analyses were performed when adequate. For all tests, *p* < 0.05 was considered statistically significant, whereas *p* > 0.05 was considered non-significant (NS). In addition, the “sample size effect” was evaluated for all behavioral tasks and measures of corticosterone concentrations using Hedge’s “*g*” test; a “*g*” value of 0.2 is regarded as a small effect, a “*g*” value of around 0.5 is a medium effect, whereas a “*g*” value of 0.8 or higher is considered as a large effect. These analyses have been conducted using the R statistical software v3.5.1 and the “compute.es” library.

## Results

Among the 138 withdrawn mice, the mean daily alcohol consumption (mL) was 4.12 ± 0.3 mL/mouse and the mean daily alcohol intake was 16.4 ± 0.3 g/kg. No significant between-group difference was observed [*F*(12,125) = 0.89; *p* = 0.86]. Thus, it can be assumed that the different withdrawn groups used in the present study received equivalent exposure to alcohol. In comparison, the mean daily water consumption was measured in two groups of water controls; the mean daily water consumption was 2.6 ± 0.5 mL, which differed significantly from two respective alcohol groups taken at random for statistical comparisons [*F*(3,36) = 3.12; *p* = 0.03]. Thus, it can be assumed that alcohol-treated mice were not dehydrated during alcohol exposure and at the time of experiments.

### Experiment 1: Effect of Alcohol Withdrawal on Emotional Reactivity

#### Elevated Plus Maze

**Figure 4:**
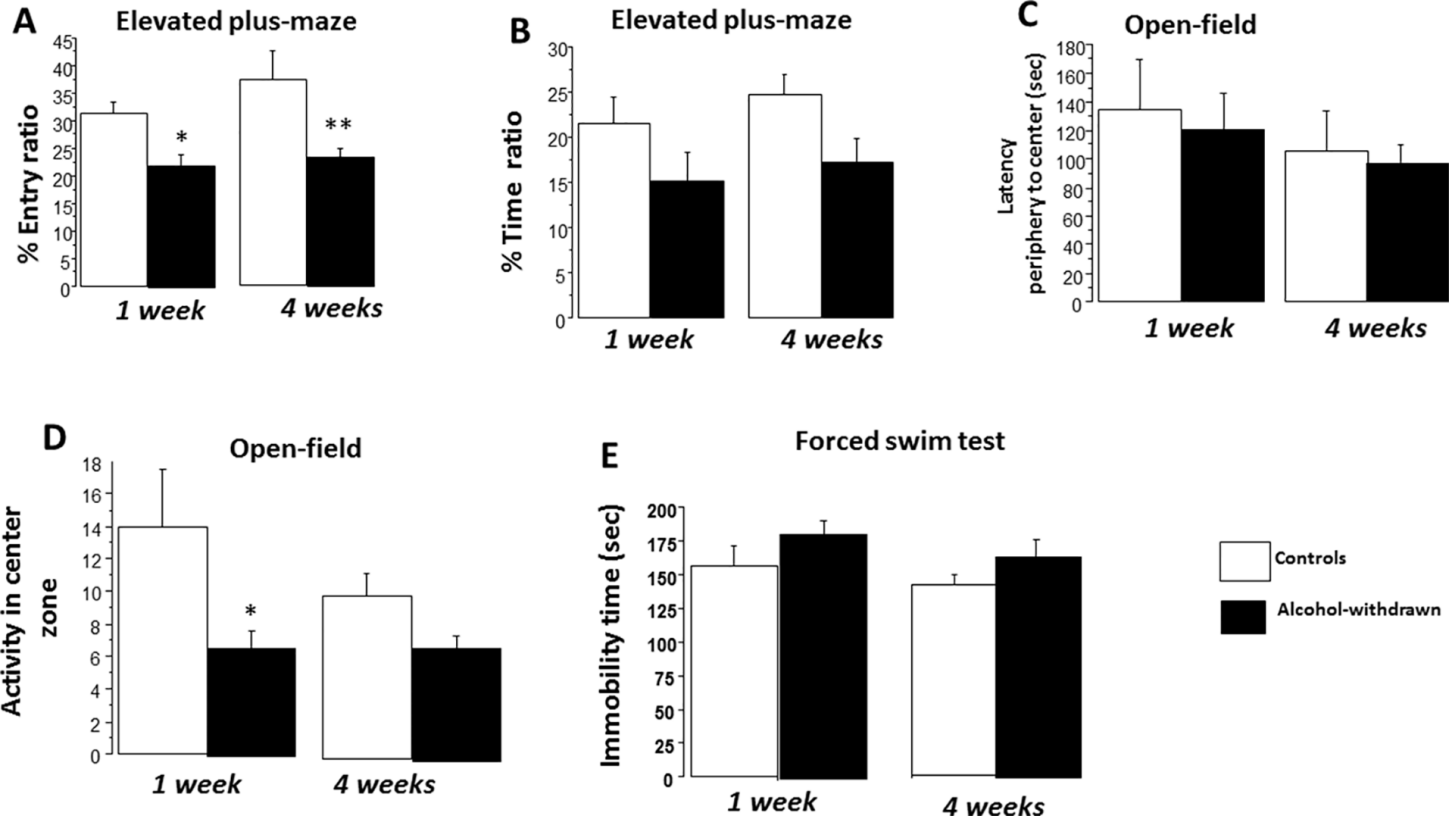
Emotional reactivity. Results are expressed as means ± SEM. ***Elevated plus maze*: (A)** One-week and 4-week withdrawn mice (*N* = 11 and 12, respectively) showed a significant decrease of entry ratio as compared to respective water controls (*N* = 12 each). **(B)** In contrast, the decrease of time ratios observed in withdrawn groups was not statistically different from respective water control groups. ***Open field*:** One-week and 4-week withdrawn groups: *N* = 9 and 12, respectively; 1-week and 4-week water controls: *N* = 10 and 11, respectively. No significant between-group difference was observed on latency to reach the periphery of the arena to the center zone. **(C)** 1W withdrawn mice showed a significant mild reduction of activity in the center zone **(D)**. ***Forced-swim test*:** One-week and 4-week withdrawn groups: *N* = 10 and 12, respectively; 1-week and 4-week water controls: *N* = 10 and 12, respectively. No significant difference was observed on the immobility time (E). *p < 0.05 and **p < 0.01 versus water controls.

The total number of entries was similar among the four groups [*F*(3,43) = 0.79; *p* = 0.66] as well as the total time spent visiting the open and closed arms of the maze [*F*(3,43) = 0.50; *p* = 0.82]. ANOVA performed on the entry ratio (**Figure 4A**) revealed a neat significant between-group difference [*F*(3,43) = 4.99; *p* = 0.004]. *Post hoc* analyses showed a significant decrease of entry ratio in withdrawn 1W mice (22.04 ± 2.17%) as compared to control 1W mice [31.32 ± 2.08%; *F*(1,21) = 9.52; *p* = 0.005; *g* = 0.82]. A decrease of entry ratio was also observed in the withdrawn 4W group (23.51 ± 1.65%) as compared to the control 4W group [37.27 ± 5.25%; *F*(1,22) = 6.22; *p* = 0.02; *g* = 1.43]. ANOVA performed on the time ratio revealed a non-significant between-group difference [*F*(3,43) = 2.48; *p* = 0.07]. A non-significant decrease of time ratio (**Figure 4B**) was observed in withdrawn 1W mice (15.22 ± 3.06%) as compared to control 1W mice [21.53 ± 2.91%; *F*(1,21) = 2.22; *p* = 0.15; *g* = 0.46]. A small significant decrease of time ratio was observed in the withdrawn 4W group (17.12 ± 2.68%) as compared to the control 4W group [24.75 ± 2.17%; *F*(1,22) = 4.87; *p* = 0.03; *g* = 0.81].

#### Open Field

ANOVA performed on the latency to reach the center zone of the arena from the periphery evidenced a non-significant between-group difference [*F*(3,38) = 0.42; *p* = 0.73]. More precisely, 1W withdrawn mice (121.5 ± 25.2 s) exhibited a comparable latency as compared to 1W control mice [134.9 ± 35.1 s; *F*(1,17) = 0.11; *p* = 0.72; *g* = 0.11]. Similarly, the 4W withdrawn group (97.4 ± 12.5 s) exhibited a latency comparable to 4W control mice [105.7 ± 28.12 s; *F*(1,21) = 0.62; *p* = 0.78; *g* = 0.14] (**Figure 4C**).

ANOVA performed on activity scores in the center zone evidenced a significant between-group difference [*F*(3,38) = 2.88; *p* < 0.05]. More precisely, withdrawn 1W mice exhibited a lower number of crossed zones as compared to control 1W mice [6.88 ± 1.18 versus 14.0 ± 3.48 respectively; *F*(1,17) = 6.21; *p* = 0.015; *g* = 0.84]. In contrast, withdrawn 4W mice (7.0 ± 0.79) exhibited non-significant reduced activity scores as compared to control 4W mice [10.0 ± 1.44; *F*(1,21) = 1.68; *p* = 0.16; *g* = 0.43] (**Figure 4D**).

#### The Forced-Swim Test

ANOVAs evidenced no significant difference on the immobility time among the four groups [*F*(3,40) = 1.77; *p* = 0.16]. More precisely, withdrawn 1W mice exhibited a non-significant higher immobility time as compared to control 1W mice [182.17 ± 10.52 s versus 156.01 ± 16.34 s, respectively; *F*(1,18) = 1.80; *p* = 0.19; *g* = 0.51]; similarly, withdrawn 4W mice exhibited a non-significant immobility time with respect to control 4W mice [163.07 ± 10.19 s versus 142.25 ± 12.12 s, respectively; *F*(1,22) = 1.87; *p* = 0.20; *g* = 0.23] (**Figure 4E**).

### Experiment 2: Alcohol-Seeking Behavior and Corticosterone Concentrations in Non-Stressed Versus Stressed Withdrawn Mice

**Figure 5:**
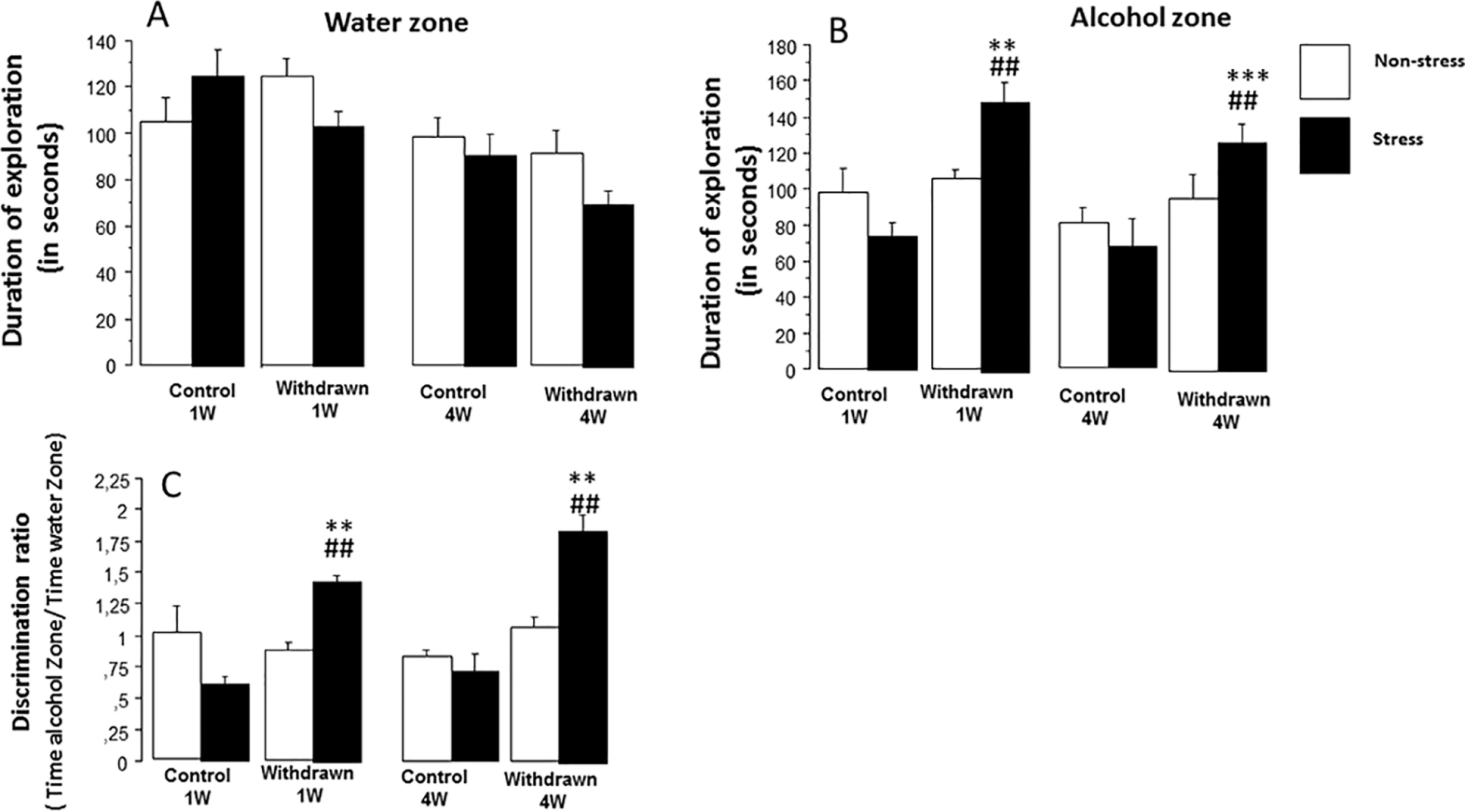
The odor recognition task. The experiment was carried out on eight independent cohorts of mice, not used in Experiment 1: stressed control 1W (*N* = 9), stressed withdrawn 1W (*N* = 8), stressed control 4W (*N* = 8), stressed withdrawn 4W (*N* = 10), non-stressed control 1W (*N* = 9), non-stressed withdrawn 1W (*N* = 9), non-stressed control 4W (*N* = 9), and non-stressed withdrawn 4W (*N* = 8). Results are expressed as means ± SEM. **(A) *Time spent in the water zone*:** No significant difference was observed between 1 week (1W) and 4 weeks (4W) non-stressed and stressed mice as compared to respective water control groups (*p* > 0.05). **(B) *Time spent in the alcohol zone*:** No significant between-group difference was observed in the non-stress condition (*p* > 0.05); in contrast, stressed withdrawn 1W and 4W groups spent significantly more time in the alcohol zone as compared to their respective water control groups (**p < 0.01 and ***p < 0.001, respectively) and as compared to non-stressed withdrawn 1W and 4W groups (^##^ *p* < 0.01 in both analyses). **(C) *Discrimination ratio*:** (time spent in the alcohol zone/time spent in the water zone): A ratio significantly above 1 means that mice exhibit a preference for the alcohol zone as compared to the water zone. No significant between-group difference was observed in the non-stress condition (*p* > 0.05); in contrast, stressed withdrawn 1W and 4W groups exhibited a significantly higher discrimination ratio as compared to their respective water control groups (**p < 0.01 in both analyses) and as compared to non-stressed withdrawn 1W and 4W groups, respectively (^##^ *p* < 0.01 in both analyses).

This experiment was performed with independent cohorts of mice not used in Experiment 1. Data are depicted in **Figure 5**.

#### Odor Recognition Task

##### Water Zone

ANOVAs evidenced a significant difference on exploration times among groups [withdrawn 1W and 4W groups and control 1W and 4W groups; *F*(3,62) = 7.08; *p* < 0.0004], a non-significant difference among conditions [stress versus non-stress; *F*(1,62) = 1.36; *p* = 0.24], and a significant interaction between groups and conditions [*F*(3,62) = 2.40; *p* = 0.075] (**Figure 5A**).

In non-stressed animals, no significant between-group difference was observed [control 1W: 105.2 ± 9.9, withdrawn 1W: 124.8 ± 7.3, control 4W: 98.4 ± 7.8, and withdrawn 4W: 91.0 ± 10.6; *F*(3,31) = 2.62; *p* = 0.067]; in contrast, a highly significant between-group difference was observed in stressed mice [control 1W: 125.2 ± 11.6, withdrawn 1W: 103.6 ± 6.6, control 4W: 81.0 ± 7.75, and withdrawn 4W: 95.87 ± 12.4; *F*(3,31) = 7.35; *p* = 0.0007], even though withdrawn 1W and 4W mice did not differ significantly from their respective stressed control groups [*F*(1,15) = 1.26 and *F*(1,16) = 2.31, respectively; *p* > 0.05 in both analyses; *g* = 0.72 and *g* = 0.84, respectively].

##### Alcohol Zone

ANOVAs evidenced a significant difference on exploration time among groups [withdrawn 1W and 4W groups and control 1W and 4W groups; *F*(3,62) = 9.9; *p* < 0.001], a non-significant difference among conditions [stress versus non-stress; *F*(1,62) = 1.7; *p* = 0.19], but a significant interaction between groups and conditions [*F*(3,62) = 4.75; *p* = 0.004] (**Figure 5B**). In the non-stress condition, no significant between-group difference was observed [control 1W: 97.7 ± 15.1, withdrawn 1W: 105.0 ± 4.54, control 4W: 81.0 ± 7.75, and withdrawn 4W: 95.87 ± 12.4; *F*(3,31) = 1.03; *p* = 0.38]. In the stress condition, a significant between-group difference was observed [control 1W: 74.6 ± 7.9 s, withdrawn 1W: 150.1 ± 11.5, control 4W: 68.3 ± 14.6, and withdrawn 4W: 127.5 ± 10.6; *F*(3,31) = 12.31; *p* = 0.0001]. The withdrawn 1W group spent a higher exploration time as compared to the control 1W group [*F*(1,15) = 4.78; *p* ≤ 0.008; *g* = 2.53], and similarly, the withdrawn 4W group spent a higher exploration time as compared to the control 4W group [*F*(1,17) = 6.02; *p* = 0.006; *g* = 1.51].

##### Discrimination Ratio (Time in Alcohol Zone/Time in Water Zone)

ANOVAs evidenced a significant difference among the groups [withdrawn 1W and 4W groups and control 1W and 4W groups; *F*(3,62) = 16.36; *p* < 0.0001], a significant difference among conditions [stress versus non-stress; *F*(1,62) = 7.3; *p* = 0.008], and a significant interaction between groups and conditions [*F*(3,62) = 12.3; *p* = 0.0001] (**Figure 5C**).

In the non-stress condition, no significant between-group difference was observed [control 1W: 1.01 ± 0.20, control 4W: 0.82 ± 0.05, withdrawn 1W: 0.87 ± 0.06, and withdrawn 4W: 0.82 ± 0.05; *F*(3,31) = 0.39; *p* = 0.75]. In the stress condition, a significant between-group difference was observed [*F*(3,31) = 32.79; *p* = 0.0001]. More precisely, the withdrawn 1W group exhibited a higher discrimination ratio as compared to the control 1W group [1.44 ± 0.05 versus 0.61 ± 0.06, respectively; *F*(1,15) = 8.79; *p* = 0.006; *g* = 4.85]; similarly, withdrawn 4W mice also exhibited a higher discrimination ratio as compared to 4W controls [1.85 ± 0.12 versus 0.72 ± 0.14, respectively; *F*(1,17) = 9.52; *p* = 0.005; *g* = 2.65]. In addition, both stressed 1W and 4W withdrawn groups also differed significantly from the respective non-stressed 1W and 4W withdrawn groups [*F*(1, 15) = 46.3; *p* < 0.0001 and *F*(1,154) = 36.7; *p* < 0.0001, respectively; *g* = 3.23 and *g* = 2.18, respectively].

#### Plasma Corticosterone

Data are expressed in ng/mL. A significant between-group difference was observed [*F*(7,32) = 4.05; *p* = 0.0028]. More specifically, the stressed control 4W group (51.78 ± 5.99) differed significantly from the stressed withdrawn 4W group [72.53 ± 4.26; *F*(1,8) = 5.43; *p* = 0.04; *g* = 0.87], and the difference was also significant between stressed control 1W mice (42.77 ± 3.79) and stressed withdrawn 1W mice [71.61 ± 13.6; *F*(1,8) = 7.55; *p* = 0.02; *g* = 0.90].

In the non-stress condition, no significant difference was observed between 1W (32.93 ± 3.61) and 4W control groups [40.25 ± 4.33; *F*(1,8) = 0.12; *p* = 0.76]; similarly, no significant difference was observed between 1W withdrawn (40.38 ± 2.72) and 4W withdrawn (42.06 ± 1.07) groups [*F*(1,8) = 0.32; *p* = 0.58; *g* = 0.31].

In addition, stressed controls 1W did not significantly differed from non-stressed controls 1W [*F*(1,8) = 4.85; *p* = 0.09], whereas the difference was significant between non-stressed control 4W and stressed control 4W groups [*F*(1,8) = 6.23; *p* = 0.04; *g* = 0.87].

### Experiment 3: Baclofen but Not Diazepam Suppressed the Alcohol-Place Preference and Normalized Corticosterone Concentrations in Stressed Withdrawn Mice

#### Measurement of Diazepam and Baclofen Concentrations in Blood

Data are summarized in **Table 1**. The blood concentrations of baclofen and diazepam and their active metabolites oxazepam and nordiazepam were measured 1, 24, and 48 h after the last injection. Small concentrations were detected at the 1-h point but not at the 24-h and 48-h points. Thus, these data discard the possibility that alcohol-place preference scores are influenced by an acute effect of the pharmacological compounds.

**Table 1:** Mean concentrations (in ng/mL) of baclofen and diazepam, and their active metabolites oxazepam and nordiazepam (*N* = 3 subjects per sampling time) by LC-MS-MS technique. Baclofen, diazepam, and nordiazepam were detected and quantified in the blood of animals 1 h after the last injection, but their concentrations were below the limit of quantification (<LOQ) for the 24- and 48-h points.

		Compounds in ng/ml				
		Baclofen	Diazepam	Oxazepam	Nordiazepam	
Sample 1	Diazepam	****	648	<LOQ	12,8	1hour
Sample 2	Diazepam	****	572	<LOQ	8,2	1hour
Sample 3	Diazepam	****	532	<LOQ	6,5	1hour
Sample 4	Baclofen	4400	****	****	****	1hour
Sample 5	Baclofen	1500	****	****	****	1hour
Sample 6	Baclofen	1730	****	****	****	1hour
Sample 7	Diazepam	****	<LOQ	<LOQ	<LOQ	24 hours
Sample 8	Diazepam	****	<LOQ	<LOQ	<LOQ	24 hours
Sample 9	Diazepam	****	<LOQ	<LOQ	<LOQ	24 hours
Sample 10	Baclofen	<LOQ	****	****	****	24 hours
Sample 11	Baclofen	<LOQ	****	****	****	24 hours
Sample 12	Baclofen	<LOQ	****	****	****	24 hours
Sample 13	Diazepam	****	<LOQ	<LOQ	<LOQ	48 hours
Sample 14	Diazepam	****	<LOQ	<LOQ	<LOQ	48 hours
Sample 15	Diazepam	****	<LOQ	<LOQ	<LOQ	48 hours
Sample 16	Baclofen	<LOQ	****	****	****	48 hours
Sample 17	Baclofen	<LOQ	****	****	****	48 hours
Sample 18	Baclofen	<LOQ	****	****	****	48 hours

*** Not applicable.

#### Odor Recognition Task

Means ± SEM are depicted in **Table 2**.

**Table 2:** Time spent (in seconds) in the water and alcohol zones in withdrawn and control groups of Experiment 3. Data are expressed as means ± SEM. No significant difference was observed between groups on the time spent exploring the water zone in stressed or non-stressed water controls. In contrast, in the stress condition, vehicle-withdrawn mice spent more time exploring the alcohol zone and less time exploring the water one as compared to stressed vehicle-water controls (***p < 0.001 and *p < 0.05, respectively). Stressed withdrawn diazepam and stressed withdrawn baclofen mice spent more time in exploring the water zone as compared to stressed withdrawn vehicles (^##^ *p* < 0.01 in both analyses).

Conditions	Non-Stress	Non-Stress	Stress	Stress
Groups	Water zone	Alcohol zone	Water zone	Alcohol zone
Control-vehicle	103.8±13.0	87.8±4.3	94.7±9.8	66.2±10.7
Control-diazepam	128.3±12.4	85.8±7.4	138.6±11.1	93.7±6.3
Control-baclofen	140.5±8.2	90.7±10.8	125.8±8.3	81.7±10.6
Withdrawm-vehicle	96.5±13.4	92.8±12.4	61.1±4.0*	124.7±14.4***
Withdrawn-diazepam	144.14±9.4	116.2±14.7	98.0±14.8 ##	122.7±12.3
Withdrawn-baclofen	117.5±9.9	92.1±10.2	119±15.2 ##	105.1±17.4

##### Water Zone

**Figure 6:**
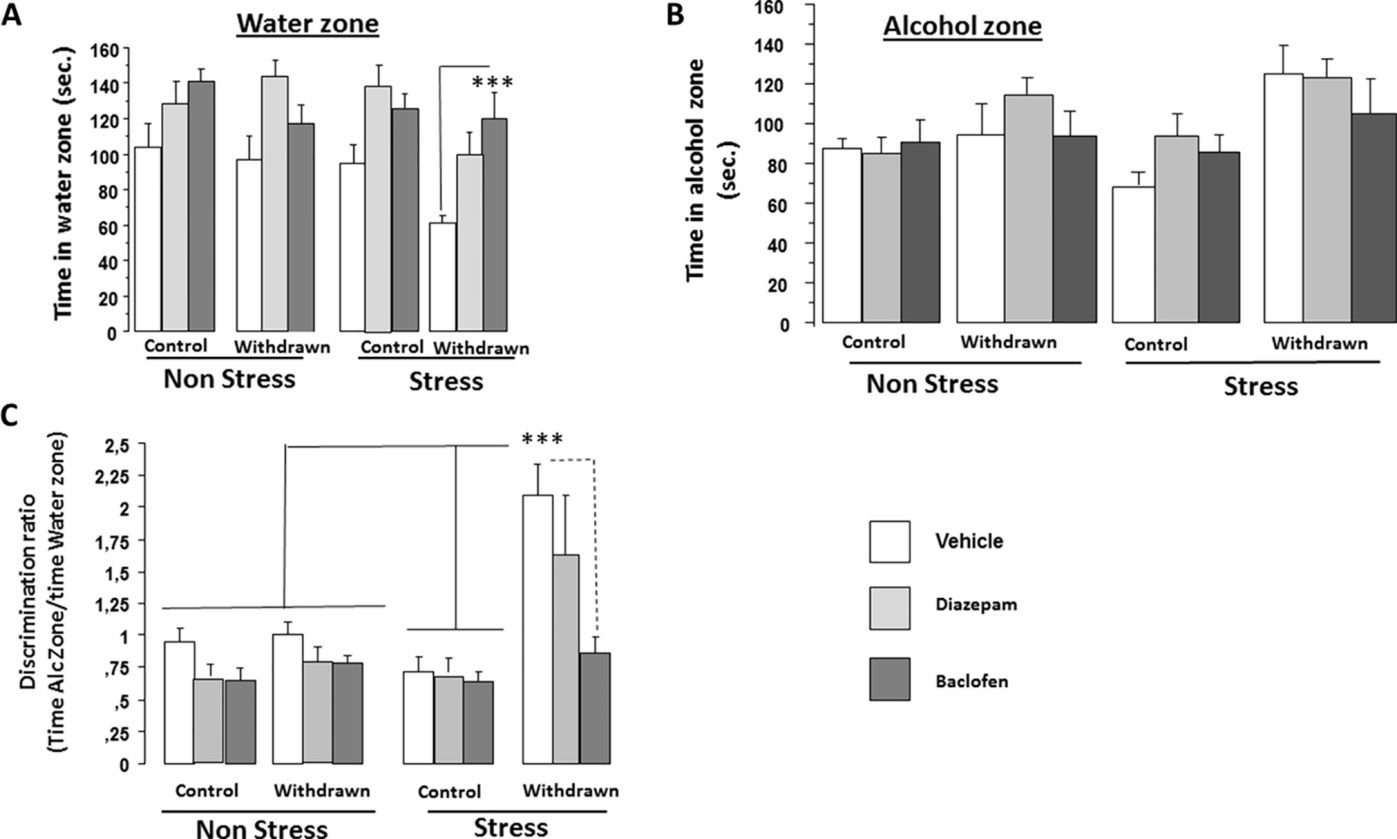
Pharmacological experiment. Experiment 3 was carried out on independent groups not used in Experiment 2 as follows: Vehicle-withdrawn mice (*N* = 9 and *N* = 8 in stress and non-stress conditions, respectively), diazepam-treated withdrawn mice (*N* = 7 in both stress and non-stress conditions), and baclofen-treated withdrawn animals (*N* = 7 in both stress and non-stress conditions) were compared to control mice also submitted to repeated injections of either vehicle (*N* = 10 in both stress and non-stress conditions), diazepam (*N* = 8 in both stress and non-stress conditions), or baclofen (*N* = 9 and *N* = 8 in stress and non-stress conditions, respectively). Results are expressed as mean ± SEM. ***Time spent in the water zone* (A):** no significant between-group difference was observed in non-stressed groups (*p* > 0.05 in all analyses); in contrast, in stressed groups, baclofen-treated withdrawn mice exhibited a higher exploration time in the water zone as compared to vehicle-treated withdrawn mice (***p < 0.001). ***Time spent in the alcohol zone* (B):** no significant between-group difference was observed in both non-stress and stress conditions (*p* > 0.05). ***Discrimination ratio* (C)** (time spent in the alcohol zone/time spent in the water zone): Vehicle-treated withdrawn mice exhibited a significantly higher discrimination ratio as compared to all groups (***p < 0.001 in all comparisons) except diazepam-stressed withdrawn mice.

Significant difference on exploration time was found among Categories [control versus withdrawn; *F*(1,86) = 5.84; *p* < 0.017], Conditions [stress versus non-stress; *F*(1,86) = 5.60; *p* = 0.02], and Treatments [vehicle, diazepam, and baclofen; *F*(2,86) = 15.65; *p* < 0.0001], but interactions between “Treatments and Conditions” [*F*(2,86) = 0.54; *p* = 0.48], “Treatments and Categories” [*F*(2,86) = 0.14; *p* = 0.86], and “Treatments × Categories × Conditions” [*F*(2,86) = 2.48; *p* = 0.08] were not significant (**Figure 6A**).

##### Alcohol Zone

ANOVAs evidenced a significant difference on exploration time among Categories [control versus withdrawn; *F*(1,86) = 14.59; *p* = 0.0003], but not among Conditions [stress versus non-stress; *F*(1,86) = 0.61; *p* = 0.43] and Treatments [vehicle, diazepam, and baclofen; *F*(2,86) = 1.56; *p* < 0.21]. The interaction between Categories and Conditions was found significant [*F*(1,86) = 4.02; *p* = 0.05] (**Figure 6B**).

In the non-stress condition, we observed no significant difference between Categories [control versus withdrawn; *F*(1,42) = 2.23; *p* = 0.14] and a non-significant Treatments effect [vehicle, diazepam, and baclofen; *F*(2,42) = 0.67; *p* = 0.51], and the interaction between Categories and Treatments was also not significant [*F*(2,42) = 1.18; *p* = 0.31]. In contrast, in the stress condition, we observed a highly significant difference between Categories [control versus withdrawn; *F*(1,44) = 13.93; *p* = 0.0005] and a non-significant Treatments effect [vehicle, diazepam, and baclofen; *F*(2,44) = 0.91; *p* = 0.40], and the interaction between Categories (water versus withdrawn) and Treatments (vehicle, diazepam, and baclofen) was also not significant [*F*(2,44) = 1.21; *p* = 0.30].

##### Discrimination Ratio (Time in Alcohol Zone/Time in Water Zone)

ANOVAs evidenced a significant difference among Categories [control versus withdrawn; *F*(1,86) = 24.05; *p* < 0.0001], Conditions [stress versus non-stress; *F*(1,86) = 9.07; *p* = 0.0034], and Treatments [vehicle, diazepam, and baclofen; *F*(2,86) = 7.78; *p* < 0.0008], and a significant triple interaction (Categories × Treatments × Conditions) was also found [*F*(2,86) = 3.59; *p* = 0.03] (**Figure 6C**).

*In the non-stress condition*, we observed no significant difference between Categories [control versus withdrawn; *F*(1,42) = 1.47; *p* = 0.23] and a significant Treatments effect [vehicle, baclofen, and diazepam; *F*(2,42) = 4.23; *p* = 0.021], but the interaction between Categories (control versus withdrawn) and Treatments (vehicle, baclofen, and diazepam) was not significant [*F*(2,42) = 0.84; *p* = 0.91; control vehicles: 0.95 ± 0.10, control diazepam: 0.71 ± 0.086, control baclofen: 0.65 ± 0.087, withdrawn vehicles: 1.0 ± 0.09, withdrawn diazepam: 0.82 ± 0.12, and withdrawn baclofen: 0.78 ± 0.06].

In contrast, *in the stress condition*, we observed highly significant differences between Categories [control versus withdrawn; *F*(1,44) = 24.16; *p* < 0.0001], Treatments [vehicle, diazepam, and baclofen; *F*(2,44) = 5.09; *p* = 0.010], and the interaction between Categories and Treatments was also significant [*F*(2,44) = 4.02; *p* = 0.024; control vehicles: 0.71 ± 0.12, control diazepam: 0.72 ± 0.09, control baclofen: 0.64 ± 0.78, withdrawn vehicles: 2.09 ± 0.24, withdrawn diazepam: 1.65 ± 0.45, and withdrawn baclofen: 0.86 ± 0.12].

More specifically, stressed withdrawn vehicles exhibited a significant higher discrimination ratio as compared to stressed control vehicles [*F*(1,17) = 24.3; *p* = 0.001; *g* = 2.26], stressed control diazepam [*F*(1,15) = 25.6; *p* = 0.001; *g* = 2.27], and stressed control baclofen [*F*(1,16) = 19.25; *p* = 0.001; *g* = 2.51] groups, as compared to stressed withdrawn baclofen mice [*F*(1,14) = 21.6; *p* = 0.001; *g* = 1.94]. In contrast, they did not significantly differed from stressed withdrawn diazepam mice [*F*(1,14) = 0.81; *p* = 0.38; *g* = 0.43].

#### Plasma Corticosterone Assay

Corticosterone concentrations are expressed in ng/mL. Corticosterone concentrations were measured in subgroups of mice (*N* = 7) chosen at random after behavioral testing. A significant difference was observed between Categories [control versus withdrawn; *F*(1,72) = 9.55; *p* = 0.0028] and Conditions [stress versus non-stress; *F*(1,72) = 65.13; *p* < 0.0001]; there is no significant Treatments effect [vehicle, diazepam, and baclofen; *F*(2,72) = 2.03; *p* = 0.13], but the triple interaction between Categories × Conditions × Treatments was found significant [*F*(2,72) = 4.56; *p* = 0.013].

The stress-induced increase of corticosterone concentrations is of greater magnitude in withdrawn mice [from 58.58 ± 2.48 to 101.32 ± 14.19 in non-stress versus stress conditions, respectively; +42.2%; *F*(1,12) = 8.79; *p* = 0.01] as compared to controls [from 53.89 ± 2.73 to 75.22 ± 4.63 in non-stress versus stress conditions, respectively; +28.8%; *F*(1,12) = 15.7; *p* = 0.002]; in addition, stressed control mice (75.22 ± 4.63) exhibited a lower corticosterone concentration as compared to stressed withdrawn mice [101.32 ± 14.19; *F*(1,12) = 5.01; *p* < 0.05; *g* = 0.87], which is not observed in the non-stress condition [*F*(1,12) = 1.61; *p* = 0.22; *g* = 0.64].

Interestingly, the stress-induced increase of corticosterone was not significant in withdrawn mice receiving baclofen [non-stress: 63.17 ± 3.84 versus stress: 70.64 ± 3.65; *F*(1,12) = 1.98; *p* = 0.18; *g* = 0.70] but was still significant in diazepam-treated withdrawn mice [non-stress: 53.77 ± 3.55 versus stress: 92.91 ± 5.43; *F*(1,12) = 36.34; *p* = 0.001]. In addition, withdrawn mice receiving baclofen did not differ significantly from those receiving diazepam in the non-stress condition [*F*(1,12) = 3.22; *p* = 0.09; *g* = 0.90] but differed significantly in the stress condition [*F*(1,12) = 11.56; *p* = 0.005; *g* = 1.7]. In control groups, baclofen and diazepam did not block the stress-induced increased of corticosterone [*baclofen*: non-stress: 47.82 ± 3.34 versus stress: 75.49 ± 4.66; *F*(1,12) = 23.20; *p* = 0.004; *g* = 2.41; *diazepam*: non-stress: 54.99 ± 4.74 versus stress: 73.09 ± 3.65; *F*(1,12) = 9.14; *p* = 0.01; *g* = 3.02].

## Discussion

Our study aims at comparing the impact of repeated injections of baclofen or diazepam during the alcohol-withdrawal period on the stress-induced alcohol-seeking behavior and HPA axis dysfunction after a long (4-week period) abstinence. The results showed that non-stressed withdrawn mice exhibited a mild increase of anxiety-like behavior in the elevated plus maze and open-field tasks while exhibiting no alcohol-place preference or alterations of circulating corticosterone concentrations as compared to water controls. In contrast, after an acute stress delivery, withdrawn mice exhibited a significant increase of alcohol-place preference and a higher circulating corticosterone concentration as compared to stressed water controls. Interestingly, repeated administration of baclofen but not diazepam during the withdrawal phase canceled out the stress-induced alcohol-place preference and normalized corticosterone levels in stressed withdrawn animals.

### Alcohol Intake

The prime issue to be addressed rested with assessing whether the motivational and endocrine alterations observed in alcohol-withdrawn mice as compared to water controls may be caused by differences in diets. Findings evidence that differences in the daily amount of food consumption may not be held accountable for the deficits since we have already demonstrated that pair-fed animals receiving an isocaloric solution of dextri-maltose during the same duration (6 months period) of alcohol exposure exhibited no memory deficits (1). In addition, our earlier findings elicited that mice still under alcohol consumption did not exhibit memory deficits or HPA axis dysfunction after the 6-month alcohol exposure period, as compared to withdrawn animals; conversely, such a pattern would occur if the diet were to be responsible for the deficits (13). Moreover, since most mice strains exhibit low appetence for alcohol, they often restrain their daily liquid intake and exhibit signs of dehydration. Such was not the case in our study, since the C57BL/6 strain is an alcohol-preferring strain (36); further, mice submitted to alcohol drank a higher daily amount of liquid solution as compared to water controls. Thus, alcohol-withdrawn mice were not dehydrated during alcohol exposure. Eventually, the daily alcohol intake during alcohol exposure in the different withdrawn groups was similar: hence, we may legitimately infer that all groups were equally exposed to alcohol, thus allowing for valuable comparisons among the different cohorts. The mean daily alcohol intake within the framework of our experiments was slightly above amounts observed in other studies involving either C57BL/6 mice (10 mg/kg/day) (36) or rats also submitted to a forced consumption diet (12.6 g/kg/day) (42).

### Emotional Reactivity, Hypothalamic–Pituitary–Adrenal Axis Dysfunction, and Alcohol-Seeking Behavior

The rationale underlying the odor recognition task is that the preference for the alcohol zone is likely linked to increased anxiety-like reactivity, induced by alcohol withdrawal (38). We showed, however, that withdrawn mice in the reference non-stress condition exhibited only a mild increase of anxiety-like reactivity in the elevated plus maze and open field; meanwhile, we found no evidence of any alcohol-place preference in the odor recognition task and normal circulating corticosterone concentrations.

The lack of severe withdrawal symptoms (such as increased anxiety and tremors) in withdrawn mice is puzzling insofar as the daily alcohol intake is relatively high. Moreover, most of the literature shows that withdrawn animals and humans exhibit potentially elevated corticosterone levels during the acute withdrawal phase and that prolonged withdrawal and abstinence are rather characterized by a blunted corticosterone response over time (6): such was not the case in our experiments since basal corticosterone levels normalized 4W after alcohol withdrawal, whereas their HPA axis became more sensitive to the effects of acute stress.

Several factors may, however, account for the discrepancies observed. Firstly, the withdrawal procedure implemented in our study was not abrupt since the amount of alcohol in the solution was gradually reduced down to water only over the 15 days of the withdrawal phase. Such a gradual withdrawal is likely to have induced a negative withdrawal impact reduction on emotional reactivity; secondly, animals were evaluated for emotional reactivity at least 1 week after withdrawal, rather than in the immediate wake of alcohol intake cessation; since we had added wooden marbles in the individual cages so as to further enrich the subjects’ environment, such a feature is likely to have alleviated anxiety in withdrawn animals. Thus, the withdrawal procedure and housing conditions account for the finding that withdrawn mice exhibit only a mild enhancement of anxiety-like reactivity in the elevated plus maze and open-field tasks.

Interestingly, blunted levels of circulating corticosterone are often reported after withdrawal in human or animal studies—a feature that is not observed in the reference (non-stress) condition. Our data are, however, congruent with one of our earlier studies to the effect that plasma corticosterone concentrations in naïve withdrawn mice (not submitted to behavioral testing) were similar to those of water controls (13). Several factors can account for the discrepancies between studies. Thus, we hypothesize that the enrichment of the cage used in the present study exerts a chronic stimulation of the HPA axis activity *via* increased exploration in the cage and thus normalizes the HPA axis activity in 4-week withdrawn mice. In addition, the withdrawal procedure in our study is progressive; such a procedure can reduce the neurobiological alterations often observed after an abrupt cessation of alcohol intake. Another factor can also be linked to the strain of mouse used, insofar as C57BL/6 is an alcohol-preferring strain (36) whose HPA axis responses to alcohol and stress differ from other mouse strains (43–45). Species differences may therefore further account for the discrepancies.

Moreover, it is noteworthy that withdrawn animals exhibit a higher sensitivity of HPA axis to stress, as compared to controls. We reported previously (13) that withdrawn animals exhibited, however, sustained and exaggerated stress-induced increases of corticosterone into the hippocampus and the prefrontal cortex. The prefrontal cortex and the hippocampus exert a negative feedback on HPA axis activity (46, 47), partly *via* the GC receptor (48). Thus, the persistent alterations of corticosterone rises after stress in these brain areas can reduce their negative feedbacks on the HPA axis activity, contributing thereby to higher circulating corticosterone levels in withdrawn mice, as compared to stressed controls.

Interestingly, the emergence of alcohol-seeking behavior in stressed withdrawn mice is associated with an exaggerated increase of plasma corticosterone concentration. Since alcohol-seeking behavior in the odor recognition task did not involve a direct alcohol intake, and since alcohol withdrawn mice exhibited no spontaneous preference for the alcohol zone in the non-stress condition, we hypothesized therefore that stress likely re-activates the memory of an anxiolytic state and/or of the rewarding effects of alcohol, leading to the emergence of an alcohol-seeking behavior. This hypothesis remains however speculative and requires as such additional experiments for further, extended substantiation.

### Baclofen but Not Diazepam Cancels Out the Stress-Induced Alcohol-Seeking Behavior and Hypothalamic–Pituitary–Adrenal Axis Disorders in Withdrawn Mice

We reported here-above that repeated diazepam administration did not counteract the stress-induced motivation for alcohol and neuroendocrine disorders in withdrawn 4W mice. We previously evidenced corrective effects of a similar repeated diazepam administration on working memory alterations after a short (1 week) but not a long (6 weeks) withdrawal period (13). We hypothesized that the failure of diazepam to reverse the cognitive dysfunction in 6-week withdrawn mice could stem either from persistent alterations of GABA_A_ receptors (24, 49) or other alcohol-induced neuroadaptations that progressively developed over time (14, 16). Indeed, chronic exposure to alcohol produces brain adaptive changes in several neurotransmitter systems, including GABA, glutamate, and norepinephrine pathways (26) in order to compensate for alcohol-induced destabilization and restore neurochemical equilibrium (50). In particular, in different rodent models of alcohol addiction, a reduction in number, function, and sensitivity to GABA of the GABA_A _receptors have been reported (49, 51–54) as well as alterations of plasticity between synaptic and extrasynaptic receptors (55, 56). These alterations can, in turn, reduce the efficacy of diazepam to counteract the protracted alterations of the HPA axis activity in withdrawn mice.

In sharp contrast, baclofen suppressed the alcohol-place preference and HPA axis disorders in stressed withdrawn mice. Baclofen is an agonist of GABA_B_ receptors and, as such, has exhibited to date its efficacy in alcohol relapse prevention (25, 57, 58). Baclofen was found to reduce alcohol intake in rodent drinking models (29, 59–62) as well as motivation to alcohol (62–66). The reducing effect of baclofen on ethanol intake has been found to be enantioselective and bidirectional (67, 68). In the present study, however, baclofen is not detectable in the blood of animals at the time of behavioral testing: therefore, its beneficial impact cannot be ascribed to its acute effects.

Interestingly, stressed withdrawn mice treated with baclofen were found to increase the exploration of the water zone, while still exploring the alcohol zone at the same level as stressed vehicles. Thus, whereas stressed withdrawn vehicle mice explored almost exclusively the alcohol zone, and exhibited a strong cue reinstatement of alcohol-seeking behavior, withdrawn animals receiving baclofen were able to shift from one zone to the other: thus, they exhibited a restoration of a cognitive/motivational flexibility that was not observed in diazepam-treated withdrawn mice or in vehicle-treated subjects. The enhancement of exploratory behavior observed in stressed withdrawn mice receiving baclofen cannot be ascribed to side effects on sensorimotor processes, since baclofen did not induce perseveration or hyper-activity in controls or in non-stressed withdrawn mice. As suggested by de Beaurepaire (69), baclofen exerts neuro-modulatory effects on several neurotransmitter systems and signaling pathways, which can alter the processing of stress and cue in the reward network and, ultimately, the functional connectivity within this network in such a way that cues associated with reward lose their meaning. Our findings remain congruent with earlier studies to the effect that baclofen attenuates the cue-induced reinstatement of alcohol-seeking behavior in alcohol-preferring rats (65). Overall, our present findings are congruent with those reported by Geisel et al. (70) who evidenced in abstinent alcoholics increased plasma GC levels, which were decreased in baclofen-treated patients, up to 14 weeks after treatment.

## Conclusion

Overall, we aimed at providing evidence that in our experimental conditions, acting on the GABA_B_ receptor during the alcohol-withdrawal phase through repeated baclofen administration rather than diazepam counteracted more so the protracted hyper-reactivity of HPA axis to stress and alcohol-seeking behavior in stressed withdrawn male mice. Since several studies evidenced different HPA axis responses to alcohol consumption and withdrawal in female as compared to male rats (71, 72), our present findings may indeed be restricted to male mice. Thus, an extended development to our current study would involve the following stakes, namely, to determine whether similar alcohol-induced endocrine and motivational disorders are to be observed in female mice and, further, to assess whether baclofen and diazepam bear similar counteracting effects on such disorders.

## Ethics Statement

This study was carried out in accordance with the recommendations of the EU Directive 2010/63/EU for animal experiments and by the local ethical committee of Bordeaux (#5012089).

## Author Contributions

YR and NH were involved in behavioral and neurobiological studies and data analyses. MC and XN were involved in statistical analyses. DB was involved in the experimental design and writing of the paper. All authors have approved the final article.

## Funding

Except for income received from primary employers, no financial support or any compensation has been received from either any individual or corporate entity over the past 3 years for either research or professional service in relation with this study. Further, no single personal financial holding may exist or be perceived as constituting a potential conflict of interest.

## Conflict of Interest Statement

The authors declare that the research was conducted in the absence of any commercial or financial relationships that could be construed as a potential conflict of interest.
